# Prediction of the Clinical Outcomes of Sigmoid Volvulus by Abdominal X-Ray: AXIS Classification System

**DOI:** 10.1155/2018/8493235

**Published:** 2018-11-15

**Authors:** Rei Ishibashi, Ryota Niikura, Nobuya Obana, Sho Fukuda, Mayo Tsuboi, Tomonori Aoki, Shuntaro Yoshida, Atsuo Yamada, Yoshihiro Hirata, Kazuhiko Koike

**Affiliations:** ^1^Department of Gastroenterology, Graduate School of Medicine, The University of Tokyo, Tokyo, Japan; ^2^Department of Gastroenterology, Osaki Citizen's Hospital, Miyagi, Japan; ^3^Endoscopy and Endoscopic Surgery, The University of Tokyo Hospital, Tokyo, Japan; ^4^Division of Advanced Genome Medicine, The Institute of Medical Science, The University of Tokyo, Tokyo, Japan

## Abstract

**Aim:**

Early diagnosis and evaluation of the severity of sigmoid volvulus are necessary for management and early intervention. We developed a new predictive classification system for sigmoid volvulus based on X-ray findings.

**Methods:**

We retrospectively analyzed 66 patients diagnosed with sigmoid volvulus using the electronic medical records at the Osaki Citizen's Hospital and the University of Tokyo Hospital from 2008–2015. We classified patients according to the coffee-bean sign mesenteric axis on X-ray (AXIS classification: group A, 0–90°; group B, 90–135°; and group C, >135°). We examined the association between AXIS classification and severe sigmoid volvulus, intestinal necrosis, need for surgery, 30-day mortality, and length of stay using the Cochran–Armitage trend test.

**Results:**

In total, 66 patients were analyzed. They had a mean age of 76.9 years, and 47 (71.0%) were male. They were classified into three groups according to the AXIS classification system (group A, 40 patients; group B, 23 patients; and group C, 3 patients). Group C had a significantly higher frequency of severe sigmoid volvulus (100%) compared to group B (30%) and group A (15%). AXIS classification was significantly associated with the severity of sigmoid volvulus (*p* = 0.003), necrosis (*p* = 0.004), and need for surgery (*p* = 0.001), but not with the 30-day mortality or the length of stay.

**Conclusions:**

We developed the AXIS classification system to predict the severity of sigmoid volvulus. This new classification system may facilitate triage and therapeutic decision-making for sigmoid volvulus patients.

## 1. Introduction

Sigmoid volvulus is an obstructive bowel disease caused by abnormal twisting of the sigmoid colon and mesentery. It is the third most common cause of acute large-bowel obstruction [1–3], and its incidence is rising due to increasing life expectancy and changes in lifestyle and dietary habits. Sigmoid volvulus begins with progressive abdominal pain, nausea, and abdominal distention and can lead to severe intestinal ischemic change, peritonitis, sepsis, and death [4, 5].

The mortality rate is 11–80% among patients with severe intestinal ischemic change and 6–24% among patients with nonsevere intestinal ischemic change [3]. Early diagnosis and intervention decrease the mortality rate, because nonsevere ischemic change is reversible at an early stage [4]. Thus, predicting the severity of sigmoid volvulus is important.

The coffee-bean sign, an inverted U-shape between the twisting and distending of the sigmoid colon and mesenteric axis [3, 6], is an X-ray finding diagnostic of sigmoid volvulus. However, its association with the severity of sigmoid volvulus is unclear. Indeed, the direction of the coffee-bean sign differs among case reports [7, 8]. Therefore, we developed a new predictive classification system based on abdominal X-ray indications of sigmoid volvulus (the AXIS classification system). The AXIS classification system categorizes sigmoid volvulus patients into three groups according to the direction of the axis of the coffee-bean sign. We evaluated the associations between the AXIS classification and the clinical outcomes of patients with sigmoid volvulus.

## 2. Materials and Methods

### 2.1. Study Design, Settings, and Patients

We retrospectively enrolled 70 patients suspected to have sigmoid volvulus using the electronic medical records of the participating hospitals. The study population included 38 patients treated at the Osaki Citizen's Hospital (Miyagi, Japan) from June 2009 to October 2014 and 32 patients treated at the University of Tokyo Hospital (Tokyo, Japan) from January 2008 to March 2015.

Four patients were excluded because of a change in diagnosis to simple constipation or ileus based on imaging findings, so 66 patients were included in the analyses. The study protocol was approved by the Institutional Review Board of the University of Tokyo Hospital.

### 2.2. Diagnosis of Sigmoid Volvulus

Sigmoid volvulus was diagnosed based on symptoms (distension and pain) and radiographic findings, i.e., coffee-bean sign on abdominal X-ray.

### 2.3. AXIS Classification

**Figure 1:**
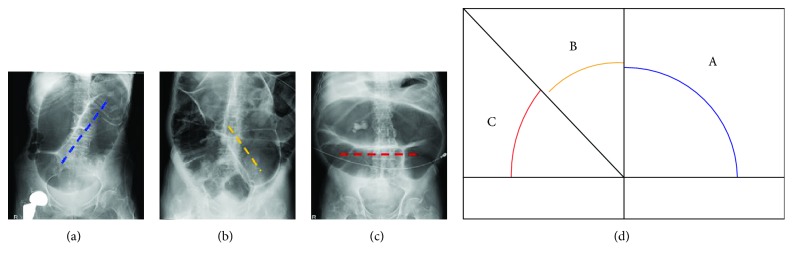
X-ray images of AXIS group A (a), group B (b), and group C (c). Diagrams of AXIS group A (0–90°), group B (90–135°), and group C (>135°) (d).

Patients were categorized into three groups according to the coffee-bean sign mesenteric axis. With the spine as the vertical axis, AXIS group A was defined to have a coffee-bean sign mesenteric axis of 0–90° from the horizontal, group B was defined to have 90–135°, and group C was defined to have >135° (Figure 1).

### 2.4. Treatment

Sigmoid volvulus was treated according to the degree of abdominal symptoms and severity of intestinal ischemia. In cases without severe abdominal pain or severe intestinal necrosis, rehydration therapy with fasting, flexible endoscopic detorsion, or intubation of an endoscopic sliding tube (ST-03 40 cm, Olympus Optical, Tokyo, Japan) was performed by endoscopists using an electronic video endoscope (PCF-260AI, Olympus Optical, Tokyo, Japan). If these therapies failed, open sigmoidectomy was performed. Open sigmoidectomy was performed for patients diagnosed with, or suspected of having, peritonitis, sepsis, or severe intestinal necrosis.

### 2.5. Outcomes and Variables

The outcomes evaluated were (i) severe sigmoid volvulus; (ii) severe intestinal necrosis, defined as an irreversible ischemic change diagnosed by endoscopic findings or pathological findings of surgical specimens; (iii) need for surgery; (iv) length of stay; and (v) 30-day mortality. Severe sigmoid volvulus was defined as any of intestinal necrosis, need for surgery, and 30-day mortality.

We also evaluated age, sex, performance status (PS) [9], American Society of Anesthesiologists physical status (ASA score) [10], body mass index (BMI), and comorbidities (using the Charlson comorbidity index (CCI)) [11].

### 2.6. Statistics

We evaluated the association between the AXIS classification and severe sigmoid volvulus, intestinal necrosis, need for surgery, length of stay, and 30-day mortality using the Cochran–Armitage test for trend. A *p* value < 0.05 was considered indicative of statistical significance.

The intra- and interobserver agreement of RI and between RI and RN was analyzed using kappa values as follows: >0.80, excellent agreement; >0.60–0.80, good agreement; >0.40–0.60, moderate agreement; >0.20–0.40, fair agreement; and ≤0.20, poor agreement. Statistical analyses were performed using STATA 13 software (StataCorp, College Station, TX).

## 3. Results and Discussion

Of the 66 patients, 40, 23, and 3 were classified into AXIS groups A, B, and C, respectively. Sixty-five patients underwent abdominal X-ray imaging in the anteroposterior supine view, and one patient had it in the anteroposterior standing view.  Table 1 shows the patient characteristics. Their mean age was 76.9 years, and 47 (71.0%) were male. Fifty-seven (86.4%) patients had an ASA score ≥ 2, and 29 (43.9%) had a CCI ≥ 2. Of the 66 patients, 1 patient was treated with rehydration therapy; 51 patients underwent only endoscopic interventions, such as flexible endoscopic detorsion or endoscopic placement of a sliding tube; 5 patients underwent endoscopic detorsion and subsequent sigmoidectomy; and 9 patients received a sigmoidectomy without endoscopic interventions. Of the 14 patients who underwent sigmoidectomy, 7 patients received a primary anastomosis without a stoma and 1 patient had a primary anastomosis with a stoma. Six patients were treated using Hartmann's procedure. Of the patients who received surgery, two had complications: aspiration pneumonitis and rectal fistula. These complications were resolved by conservative treatment.

Table 2 shows the associations between the AXIS classification and clinical outcomes. AXIS group C had a significantly higher proportion of severe sigmoid volvulus (100%) compared to group B (30%) and group A (15%). AXIS classification was significantly associated with severe sigmoid volvulus (*p* = 0.003, for trend), intestinal necrosis (*p* = 0.004, for trend), and need for surgery (*p* = 0.003, for trend), but not with length of stay or 30-day mortality.

The intraobserver agreement for AXIS classification was excellent (agreement: 93.9%, mean *κ* = 0.881), and the interobserver agreement was good (agreement: 78.8%, mean *κ* = 0.612).

The X-ray coffee-bean sign is diagnostic in 60–80% of sigmoid volvulus cases [1, 4, 12]. However, its utility in predicting the severity of sigmoid volvulus is unclear. In this study, we developed the AXIS classification system, which was significantly associated with severe sigmoid volvulus, including intestinal necrosis.

In all AXIS group C patients, the sigmoid colon was twisted and its origin was necrosis on operative findings. These findings suggest that torsion interrupts mesenteric blood flow and results in intestinal necrosis [13, 14]. The severity of sigmoid volvulus may depend on the length of time after the onset of sigmoid twisting. In our additional analyses (Supplementary Table 1), the time after onset was longer in AXIS group C. With increasing time after onset, the distended sigmoid colon might change the direction of the sigmoid colon's axis, given that the descending colon and rectum are fixed in the retroperitoneal space.

AXIS classification was significantly associated with the intestinal necrosis and need for surgery, but not with the mortality rate. Only one of the 66 patients died during the study period, which is lower than the number of patients in prior reports [1, 3, 15, 16]. AXIS classification enabled early diagnosis, intensive treatment, and surgeries with few complications. However, the small sample size hampered the evaluation of the mortality rate.

With the AXIS classification system, a simple abdominal X-ray performed in the emergency room can be used to predict the severity of sigmoid volvulus. The high rates of intra- and interobserver agreement demonstrated its ability to facilitate triage and aid decisions about whether patients should be transferred to a high-volume medical center.

Various parameters have been used to evaluate sigmoid volvulus, but to date these have not included X-ray findings.

Indeed, the duration of clinical symptoms and the rapidity of colonic twisting do not enable the evaluation of the prognosis of sigmoid volvulus [4, 17, 18]. The Atamanalp classification system, which is based on bowel condition and endoscopic findings, is used for treatment decisions and prognosis estimation [18]. However, in contrast to the AXIS classification system, it cannot predict severity during the early phase of sigmoid volvulus.

Our study had several limitations. First, it had a retrospective design. The treatment strategy, including an assessment of the need for surgery, was based on an evaluation of the patient's condition, the results of imaging examinations, and the opinions of the attending physician, endoscopist, and surgeon. This treatment strategy may have influenced the clinical outcome. Second, our study population was relatively small, such that predictive factors may have been missed. Finally, we did not validate the predictive efficacy of the AXIS classification system in a second patient population.

## 4. Conclusions

The AXIS classification system enables the prediction of the severity of sigmoid volvulus and facilitates treatment decision-making at an early stage of the disease.

## Figures and Tables

**Table 1:** Patient characteristics.

Variable	AXIS classification			
	Total *n* = 66 (%)	Group A *n* = 40 (%)	Group B *n* = 23 (%)	Group C *n* = 3 (%)
Age, years (±SD)	76.9 ± 10.3	78.3 ± 8.1	74.0 ± 13.4	80.3 ± 4.0
Sex, male	47 (71.2)	27 (67.5)	18 (78.3)	2 (66.7)
Performance status score ≥ 2	42 (63.4)	22 (55.0)	18 (78.3)	2 (66.7)
ASA score ≥ 2	57 (86.4)	32 (80.0)	22 (95.7)	3 (100)
Body mass index (±SD)	20.1 ± 4.3	21.4 ± 4.4	20.1 ± 4.2	22.1 ± 4.7
History of abdominal surgery	18 (27.3)	13 (32.5)	5 (21.7)	0
Charlson comorbidity index ≥ 2	29 (43.9)	17 (42.5)	12 (50.2)	0
Comorbidities				
Ischemic heart disease	12 (18.2)	7 (17.5)	5 (21.4)	0
Chronic heart failure	3 (4.5)	2 (5.0)	0	1 (33.3)
Diabetes mellitus	8 (12.1)	4 (10.0)	3 (13.0)	1 (33.3)
Peripheral vascular disease	2 (3.0)	2 (5.0)	0	0
Cerebrovascular disease	12 (18.2)	5 (12.5)	6 (26.1)	1 (33.3)
Dementia	4 (6.1)	2 (5.0)	2 (8.7)	0
COPD	6 (9.1)	2 (5.0)	4 (17.4)	0
Liver cirrhosis	4 (6.1)	2 (5.0)	2 (8.7)	0
Chronic renal disease	4 (6.1)	2 (5.0)	2 (8.7)	0
Cancer	8 (12.1)	6 (15.0)	2 (8.7)	0

Abbreviations: ASA: American Society of Anesthesiologists; COPD: chronic obstructive pulmonary disease; SD: standard deviation.

**Table 2:** Associations between AXIS classification and clinical outcomes.

Outcome	AXIS classification			
	Group A *n* = 40 (%)	Group B *n* = 23 (%)	Group C *n* = 3 (%)	*p* for trend
Severe sigmoid volvulus^†^				
No	34 (85.0)	16 (69.6)	0 (0)	**0.003**
Yes	6 (15.0)	7 (30.4)	3 (100)	
Necrosis				
No	36 (90.0)	19 (82.6)	0 (0)	**0.004**
Yes	4 (10.0)	4 (17.4)	3 (100)	
Need for surgery				
No	35 (87.5)	17 (73.9)	0 (0)	**0.003**
Yes	5 (12.5)	6 (26.1)	3 (100)	
30-day mortality				
No	40 (100)	22 (95.7)	3 (100)	0.334
Yes	0 (0)	1 (4.3)	0 (0)	
Length of stay, day (±SD)	9.53 ± 10.2	15.4 ± 25.7	16.3 ± 7.4	0.095

^†^Defined as any of intestinal necrosis, need for surgery, and 30-day mortality rate. Bold values indicate statistical significance (*p* < 0.050). Abbreviation: SD: standard deviation.

## Data Availability

The Institutional Review Board of the University of Tokyo does not allow the participant-level data to be used in other works for ethical reasons, because the opt-out for study participants did not inform them of the secondary use of their data at other institutions. For data requests, please contact yohirata@ims.u-tokyo.ac.jp.

## Supplementary Materials

Supplementary Materials Surgical history and a past history of sigmoid volvulus were not associated with AXIS classification, and the time after onset was marginally associated with more severe AXIS classification.
