# Supplementary material for: Prediction of the Clinical Outcomes of Sigmoid Volvulus by Abdominal X-Ray: AXIS Classification System
Source: Gastroenterol Res Pract. 2018 Nov 15;2018:8493235. doi: 10.1155/2018/8493235 (PMC6276422; doi:10.1155/2018/8493235)
Supplement: Supplementary Materials — Surgical history and a past history of sigmoid volvulus were not associated with AXIS classification, and the time after onset was marginally associated with more severe AXIS classification. [file 8493235.f1.pdf]

Supplementary Table 1. Associated factors for the deviation of axis in patients underwent surgery (N=14).

| | AXIS classification | | | |
|---|---|---|---|---|
| | Group A<br>n =5 (%) | Group B<br>n =6 (%) | Group C<br>n =3 (%) | P for trend |
| Time after onset (day) | | | | 0.06 |
| 0 | 2(40) | 3(50) | 0 | |
| 1 | 2(40) | 2(33.3) | 0 | |
| 2 | 1(20) | 1(16.7) | 1(33.3) | |
| 3 | 0 | 0 | 1(33.3) | |
| 6 | 0 | 0 | 1(33.3) | |
| Surgical history, no | 5(100) | 6(100) | 3(100) | Not applicable |
| yes | 0 | 0 | 0 | |
| Past history of sigmoid volvulus, no | 5(100) | 6(100) | 3(100) | Not applicable |
| yes | 0 | 0 | 0 | |

Surgical history and a past history of sigmoid volvulus were not associated with AXIS classification, and time after onset was marginally associated with more severe AXIS classification.
